# Pernicious anemia presenting with dysphagia and melanoderma: a confusing manifestation

**DOI:** 10.2144/fsoa-2023-0176

**Published:** 2024-05-20

**Authors:** Salma Souissi, Myriam Ayari, Imen Abdellali, Rim Bourguiba, Syrine Belakhal, Taieb Jomni, Mohamed Hedi Doggui

**Affiliations:** 1 Gastroenterology Department, Internal Security Forces Hospital La Marsa, Tunis, 2070, Tunisia; 2 University of Tunis El Manar, Tunis, 1068, Tunisia

**Keywords:** dysphagia, melanoderma, pernicious anemia

## Abstract

Vitamin B12 deficiency is widely recognized as a common cause of anemia. However, symptoms such as dysphagia, melanoderma, and pancytopenia, although less frequent, can also be associated with this deficiency. We report the case of a 47-year-old Caucasian man presented with dysphagia to solids associated to high heart rate, dyspnea and melanoderma. He was diagnosed with severe anemia (hemoglobin 4 g/dl) in association with pancytopenia. Further investigation confirmed that the underlying cause was severe vitamin B12 deficiency secondary to pernicious anemia. Subsequent treatment with vitamin B12 supplements led to a significant improvement in all symptoms. A review of the existing literature corroborated the rarity of severe anemia occurring in conjunction with dysphagia and melanoderma due to B12 deficiency.

## Plain language summary

Anemia is a condition where your body does not have enough healthy red blood cells. We report the case of a 47-year-old man who presented with difficulty swallowing solid food (dysphagia), a fast heart rate, difficulty breathing (dyspnea), and changes in skin color (melanoderma). After some tests, we diagnosed the patient with severe anemia and low counts of different types of blood cells (pancytopenia). The underlying cause was a severe lack of Vitamin B12, and the specific type of anemia was called pernicious anemia. Subsequent treatment with Vitamin B12 supplements led to significant improvement. Physicians should be aware of uncommon presentations of pernicious anemia to diagnose it early, avoid unnecessary investigations and to initiate rapidly simple and efficient treatment.

## Abstract (plain language summary)

Biermer's disease, is an autoimmune disorder specific of the gastric organ.

Dysphagia, melanoderma and pancytopenia are rarely observed in vitamin B12 deficiency.

Treatment with vitamin B12 supplements drastically improves these symptoms.

Physicians should be aware of uncommon presentations of PA to diagnose it early and avoid unnecessary investigations.

Pernicious anemia (PA), known also as Biermer's disease, represents an autoimmune condition primarily affecting the gastric system. This disorder is characterized by the presence of autoantibodies that specifically target intrinsic factor and/or gastric parietal cells. Consequently, it leads to chronic fundic atrophy, hinders the absorption of vitamin B12 and results in achlorhydria [1]. General prevalence is of 2%, displaying a bimodal distribution with a predominance of elderly women (peaks of 4–5%) [2]. The diagnosis of autoimmune gastritis can be challenging and relies on the demonstration of its characteristic histopathological features and the presence of autoantibodies. Classical presentation associates neurological signs with anemia evolving insidiously, giving it the name of pernicious anemia. However, it can present with uncommon manifestations, both clinically and biologically, leading to misdiagnosis. Here in, we present a case of PA revealed by dysphagia and melanoderma responding dramatically to intramuscular vitamin B12 therapy.

## Case report

A 47-year-old Caucasian male patient arrived with a chief complaint of experiencing difficulty swallowing solid foods, which had been progressing over the course of 3 months. This issue occurred within the context of an overall decline in his health and was accompanied by episodes of vomiting. He had no other significant personal or family past medical history.

**Figure 1.**
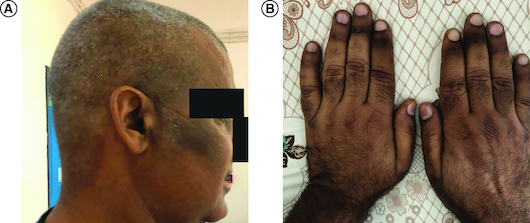
Hyperpigmentation at presentation. **(A)** Of the face, **(B)** and hands.

Upon physical examination, the patient displayed signs of conjunctival pallor, a notably elevated heart rate and dyspnea. Neurological exam revealed normal gait, intact deep tendon reflexes, and no apparent sensory deficits. Additionally, no deficits were observed in vibratory sensation or proprioception. Cutaneous examination found hyperpigmentation lesions of the face and hands related to melanoderma (Figure 1).

The investigations blood tests indicated pancytopenia (platelet = 91,000/mm^3^; leucocytes = 2500/mm^3^: neutrophils = 768/mm^3^, eosinophils = 18/mm^3^, basophils = 15/mm^3^, lymphocytes = 1585/mm^3^, monocytes = 115/mm^3^) with severe macrocytic anemia (hemoglobin = 4 g/dl, MCV = 115 μ3, MCH = 39.7 pg, MCHC = 34.7 g/dl), reticulocyte rate of 23,199/mm^3^, unconjugated hyperbilirubinemia of 38.7 μmol/l, normal renal and hepatic function. Serum cortisol and adrenocorticotrophic hormone (ACTH) showed normal results ruling out severe cortisol deficiency. Thyroid stimulating hormone was also normal.

Upper gastrointestinal endoscopy were performed and did not show suspicious oesogastroduodenal lesions explaining dysphagia, neither fundic atrophy. Histological examination of oesophageal done in order to rule out esoniphilic oesophagitis was normal. We completed then by high resolution esophageal manometry that showed no motor disorder of the esophagus.

For etiological investigation of macrocytic anemia, a bone marrow biopsy was planned. However, additional work up revealed a significant deficiency in vitamin B12 levels, measuring at 52 pg/ml, which falls well below the normal range of 126–505 pg/ml. The patient was not on vegetarian or vegan diet. Unfortunately, the measurement of serum methylmalonic acid or homocysteine were not available at our hospital, and the vitamin B12 at the tissue level was not measured neither. Meanwhile systematic fundic biopsies showed gastric corpus atrophy. So, we considered PA and completed with serum antiparietal antibodies, which come out to be positive. Anti-intrinsic factor antibodies were not available at our hospital. He, then, was diagnosed as having early-stage PA.

The patient was subsequently prescribed mecobalamin at a dosage of 1000 mg, by intramuscular route, once a week for a duration of four weeks, followed by a maintenance dose of once a month . Once his counts were improving and stable, he was discharged. His presenting symptoms were completely resolved. Two-week follow-up showed drastic improvement in pigmentation, overall well-being, normal blood count and his vitamin B12 levels improved to 300 pg/ml.

## Discussion

Vitamin B12, also known as cobalamin, plays a crucial role in the formation of hematopoietic cells. Cobalamin acts as a cofactor in various metabolic processes, including DNA synthesis, fatty acid metabolism and amino acid metabolism. Furthermore, it contributes to neuronal myelination through a mechanism that is not yet fully understood [3].

PA stands as a primary cause of vitamin B12 deficiency. It is characterized by the destruction of the gastric mucosa, leading to autoimmune atrophic gastritis, specifically type A, which results in the destruction of the oxyntic mucosa, and thus, the loss of parietal cells, which normally produce chlorhydric acid as well as intrinsic factor [4]. This deficiency, in turn, hampers the absorption of vitamin B12 by the ileum. The classical hematological manifestation of PA is macrocytic, megaloblastic anemia. Pancytopenia, a rare consequence of vitamin B12 deficiency [3], is typically associated with inefficient hematopoiesis and is observed in cases of severe and prolonged vitamin B12 deficits. The underlying mechanism behind this cytopenia is flawed DNA synthesis [5]. While all hematopoietic cell lines are affected, the impact is most profound on erythrocytes. In a study involving 201 patients with documented cobalamin deficiency, approximately two-thirds of them exhibited hematological abnormalities, with pancytopenia occurring in 5% of cases [6].

The term ‘pernicious anemia’ commonly used does not seem however to be very suitable. In fact, this condition can be diagnosed in the absence of any hematological abnormality, but often on neurological signs rather typical or other uncommon manifestations.

Indeed, it is worth noting that PA can also manifest with a variety of neurological abnormalities, without the presence of hematological features, observed in approximately 30% of cases. These neurological manifestations can vary widely, ranging from an asymptomatic state to the demyelination of white matter in the brain and spinal cord. This demyelination can manifest as dementia, peripheral neuropathy, ataxia, and less frequently, bulbar symptoms such as dysphagia. It is important to highlight that dysphagia resulting from vitamin B12 deficiency has been rarely reported in the literature [7,8].

By reviewing the literature, we found few cases that reported dysphagia as a sign revealing vitamin B12 deficiency, the first reported the case of a female patient aged 10 months who was admitted to hospital because of dysphagia, poor feeding, weakness and apathy. The final diagnosis confirmed a case of vitamin B12 deficiency anemia, attributed to the same depletion in the patient's mother. Following treatment with vitamin B12 supplements, the patient exhibited significant improvement, and all symptoms resolved [8].

Another case, reported by Edward *et al.*, involved a 66-year-old male who presented with dyspnea, productive cough, dysphagia to solids, and unintentional weight loss over the past year. Family members reported cognitive decline over 18 months, decreased concentration, and difficulties with executive functioning. The presumptive diagnosis in this case was aspiration pneumonia resulting from neurogenic dysphagia, a consequence of severe vitamin B12 deficiency associated with PA [7].

Recently, Parikh R *et al.* published a retrospective case series of five children under 2 years of age, presenting with dysphagia, megaloblastic anemia and concurrent nutritional compromise. The findings demonstrated that dysphagia could be reversed through a combination of medical nutrition therapy and injectable B12 therapy in children with severe acute malnutrition exhibiting severe megaloblastic anemia. This study highlights the prevalence of dysphagia in severe acute malnutrition cases with megaloblastic anemia and underscores its potential for reversal and cure through nutritional rehabilitation and injectable B12 therapy [9].

Neurological lesions linked to B12 deficiency are progressive and encompass a spectrum from demyelination to axonal degeneration, ultimately culminating in neuronal death. Notably, they may not always fully reverse with B12 supplementation [1]. Both human and animal studies have suggested that cobalamin deficiency can disrupt cytokine expression, which is (believed to contribute to the development of white matter myelinolytic lesions [10]. Nevertheless, neurological deficits can be reversible, especially when treatment is initiated within 6 months of initial presentation. Improvement in neurological symptoms can typically be expected within one week to three months after the initiation of supplementation [1,11,12]. The prompt administration of vitamin B12 has been shown to reverse neurological symptoms, although the degree of recovery may be influenced by the severity and duration of symptoms prior to treatment [12]. In our patient, neurogenic dysphagia completely resolved, and pancytopenia disappeared after three months of B12 supplementation.

**Figure 2.**
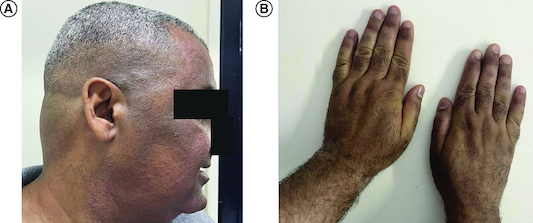
Improvement of hyperpigmentation after vitamin B12 therapy. **(A)** Of the face and **(B)** hands.

Regarding the melanoderma lesion observed in our patient, it may be attributed to vitamin B12 deficiency. This was supported by a negative screening test for Addison's disease, and the patient's symptoms notably improved after the initiation of vitamin B12 supplementation (Figure 2).

Dermatological manifestations of PA are mainly represented by glossitis, nails and hair anomalies and cases of generalized hyperpigmentation have also been reported, but localized lesion had been described in only few cases [^13-15^]. This atypical form of PA can mimic adrenal insufficiency. It has been frequently observed in black African subjects, without any other explanation. Nevertheless, despite not being recognized as a specific sign of this autoimmune disease, since no excessive dermal or epidermal melanin concentrations or infiltration of melanocytes are observed upon microscopic examination [16], the disappearance of melanoderma concurrent with the resolution of other PA-related symptoms during appropriately administered vitamin B12 therapy strongly implies the involvement of vitamin B12 deficiency.

Multiple hypotheses have been proposed to explain the mechanism by which vitamin B12 deficiency leads to hyperpigmentation. The most widely accepted would be an elevation of the blood concentration of tyrosine which is a precursor of melanin. Another hypothesis posits that a deficit exists in the transfer of melanin from the melanocytes to the keratinocytes, ultimately leading to pigment incontinence [12]. Also, the deficiency in vitamin B12 has induced, on rats on diet, the inhibition of the of the activity of hepatic tyrosine aminotransferase [17].

## Conclusion

The direct association between dysphagia and melanoderma in our patient was established as a consequence of vitamin B12 deficiency associated with PA. This confirmation was evident when all symptoms were resolved following vitamin B12 replacement therapy. The measurement of serum methylmalonic acid or homocysteine could have confirmed our findings. PA might be misdiagnosed as it causes a multifaceted disorder. Physicians should be aware of uncommon presentations of a common condition to diagnose early, avoid unnecessary investigations and to initiate rapidly simple and efficient treatment by vitamin B12 administration.
